# Targeting the PI3K/AKT pathway via GLI1 inhibition enhanced the drug sensitivity of acute myeloid leukemia cells

**DOI:** 10.1038/srep40361

**Published:** 2017-01-18

**Authors:** Hui Liang, Qi-Li Zheng, Peng Fang, Jian Zhang, Tuo Zhang, Wei Liu, Min Guo, Christopher L. Robinson, Shui-bing Chen, Xiao-Ping Chen, Fang-Ping Chen, Hui Zeng

**Affiliations:** 1 Department of Hematology, Xiangya Hospital, Central South University, Changsha, Hunan, China; 2 Department of Hematology, Third Xiangya Hospital, Central South University, China; 3 Genomic Core, Weill Cornell Medical College, NY, USA; 4 Department of Oncology, Xiangya Hospital, Central South University, China; 5 Department of Endocrinology, Xiangya Hospital, Central South University, China; 6 Department of Surgery Genomic Core, Weill Cornell Medical College, NY, USA; 7 Department of Clinical Pharmacology, Xiangya Hospital, Central South University, China; 8 Institute of Clinical Pharmacology, Central South University, China

## Abstract

Combination targeted therapy is commonly used to treat acute myeloid leukemia (AML) patients, particularly in refractory/relapse (RR) population. However, concerns have been raised regarding the safety and patient tolerance of combination chemotherapy. It is critical to choose the appropriate treatment for precision therapy. We performed genome-wide RNA profiling using RNA-Seq to compare the RR group and the complete remission (CR) group (a total of 42 adult AML patients). The Hedgehog (Hh) and PI3K/AKT pathways were upregulated in the RR population, which was further confirmed by western blot and/or qPCR. Overexpression of GLI1 in AML cells led to increased AKT phosphorylation and decreased drug sensitivity, which was attenuated by GLI1 inhibition. By contrast, neither the expression of GLI1 nor apoptosis in response to Ara-C treatment of AML cells was significantly affected by PI3K inhibition. Furthermore, co-inhibition of GLI1 and PI3K induced apoptosis of hematopoietic stem/progenitor cells (HSPCs), which raised serious concerns about the side effects of this treatment. These results indicated that GLI1 inhibition alone, but not combined inhibition, is sufficient to enhance AML drug sensitivity, which provides a novel therapeutic strategy for AML treatment.

Acute myeloid leukemia (AML) is a hematological malignancy with high incidence and recurrence rates. The high recurrence of AML reflects the incomplete eradication of leukemia stem cells by conventional chemotherapy and Ara-C combination chemotherapy, which are the most commonly used treatments. Approximately 20% of adult patients with AML fail to achieve remission with initial induction chemotherapy, and approximately 50% ultimately experience relapse after achieving complete remission^1^,^2^,^3^. A major side effect of the current chemotherapy is toxicity to hematopoietic stem cells (HSCs). Although hematopoietic stem cell transplantation (HSCT) is a promising therapeutic strategy for AML, the absence of Human leukocyte antigen(HLA)-matched donors and the risk of post-transplantation rejection are major challenges. Additionally, the high cost and mortality rate limit its application in clinical practice. As new developments have been made in molecular biology and genetics, targeted therapy or combination targeted therapy has been shown to be a promising area of research for AML^4^,^5^.

As the pathogenesis of AML involves regulation of different signalling pathways, the interaction and feedback among different signalling pathways are expected to be involved in the clinical treatment of AML. It is therefore critical to understand the interactions of these signalling pathways to provide a precise diagnosis for patients. Genomic approaches have identified somatic mutations in coding genes that are associated with patients’ prognosis. In transcriptome research, microarrays have been extensively used to identify a number of differentially expressed genes^6^,^7^,^8^. Microarray techniques and, more recently, RNA sequencing (RNA-Seq) technology have been widely used to analyse the global transcriptome changes in different malignancies^9^,^10^.

The Hh pathway plays an important role in cell proliferation, differentiation, apoptosis and migration. It has been shown to cross-talk with other signalling pathways (such as PI3K/AKT, RAS, NOTCH and others) and has been identified as a major target in the treatment of hematological malignancies^11^,^12^,^13^. In chronic myeloid leukemia (CML), inhibition of the Hh pathway could reduce the number of leukemia stem cells and reverse drug resistance to imatinib^14^. Recently Zahreddine *et al*. also demonstrated that a key transcription factor of the Hh pathway, GLI1, affects drug resistance by inducing glucuronidation, which could be reversed by a Hh inhibitor^15^. In addition, several clinical studies have reported that the expression of GLI1 is closely related to the prognosis of acute myeloid leukemia (AML)^16^.

Meanwhile, sustained activation of the PI3K/AKT pathway has been identified in different types of leukemia, and this effect promotes cell cycle progression, inhibits cell apoptosis and facilitates invasion and metastasis of cancer cells^17^,^18^,^19^. Our group found that PTEN-regulated PI3K/AKT activation is closely related to the progression and drug resistance of adult AML^20^. In addition, it has been shown that combined inhibition of the Hh and PI3K/AKT pathways has synergistic anti-leukemia effects in AML and in chronic lymphocytic leukemia^21^,^22^,^23^.

However, whether there is cross-talk between the Hh and PI3K/AKT pathways in AML drug resistance/relapse and the potential risk of combined inhibition of these pathways in AML patients are still unknown. The possibility of using Hh and PI3K double inhibition in clinical AML treatment requires further study.

By comparing the clinical samples of AML-RR and AML-CR patients using RNA-Seq, we showed that both the Hh and PI3K/AKT pathways were upregulated in the AML-RR group. Moreover, overexpression of GLI1 decreased drug sensitivity in AML cells, which could be reversed by a GLI1 inhibitor. In addition, GLI1 inhibition blocked the PI3K/AKT pathway, whereas PI3K inhibition had no effect on GLI1 expression or AML cell drug sensitivity. This study describes a novel mechanism of Hh and PI3K/AKT pathway interaction in chemo-resistance and relapse of AML patients, which provides guidance to develop an efficient and safe combination targeted therapy for AML-RR patients.

## Results

### The Hh pathway is activated in AML-RR patients

**Figure 1:**
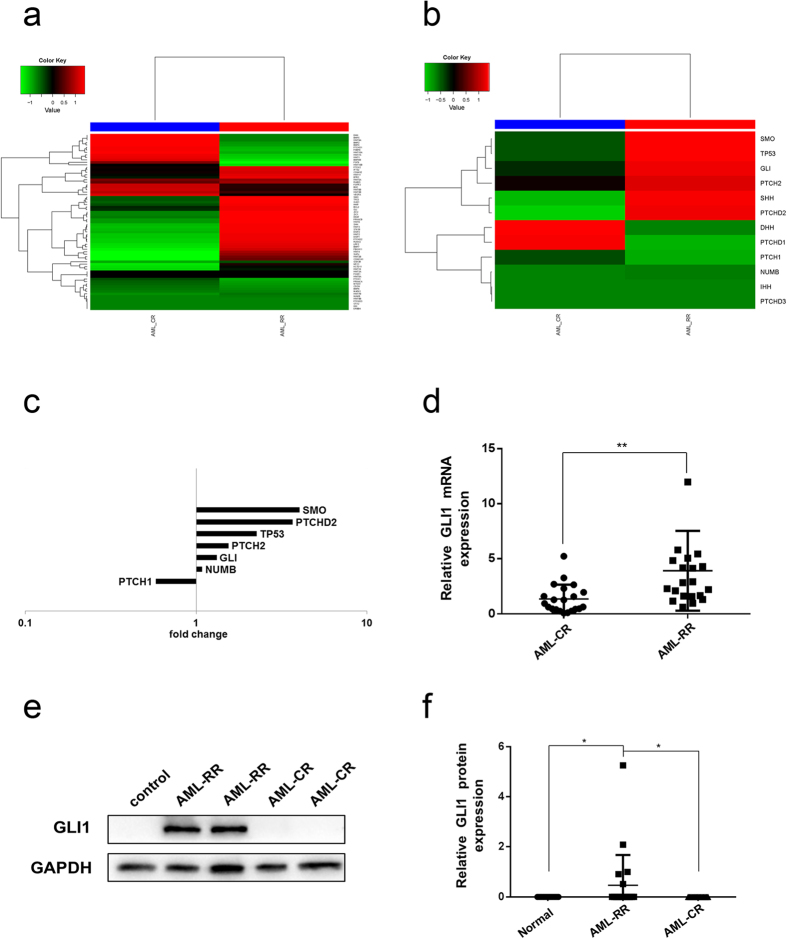
Gene expression detected by RNA-Seq and expression of GLI1 in AML patients. (**a**) Hh pathway-related gene expression in AML patients. (**b**) Core component Hh pathway gene expression in AML patients. (**c**) Fold-change of selected Hh-related genes in AML patients. (**d**) GLI1 transcript expression in AML-CR and AML-RR patients. (**e**,**f**) Western blot analysis and quantification of GLI1 protein expression in AML-CR and AML-RR patients. *P < 0.05, **P < 0.01.

Forty-two AML patients were recruited in this study. Based on their clinical history, the patients were classified into two groups: the refractory/relapse (AML-RR) group and the complete remission (AML-CR) group (Tables 1 and 2). RNAs were isolated from mononuclear cells (MNCs) of these patients and analysed using RNA-Seq. To identify significant differences in the transcriptomic profiles of the two sample groups (AML-RR and AML-CR), we generated a pair wise scatter matrix by CummeRbund. This method compares and correlates the RPKM profile of all expressed genes in the two sample groups, and it also shows the density distribution of the RPKM for the expressed genes. The RPKM of all expressed genes ranged from 0.003 to 3000 (log_10_RPKM −2.5 to 3.5), with the majority of the genes ranging from 1 to 100 (log_10_RPKM 0 to 2). The majority of genes involved in the Hh pathway, such as GLI, SMO, SHH, and PTCH, (Fig. 1a–c, Table 3) were upregulated in the AML-RR group compared with the AML-CR group. qRT-PCR and western blot further confirmed the upregulation of GLI1, a key transcript of the Hh pathway, at the mRNA (Fig. 1d, P < 0.01) and protein (Fig. 1e,f, P < 0.05) levels in the AML-RR group. These results were consistent with the RNA-Seq results, which indicated that the Hh pathway may be related to the progression and prognosis of AML.

### The PI3K/AKT pathway is also activated in AML-RR patients

**Figure 2:**
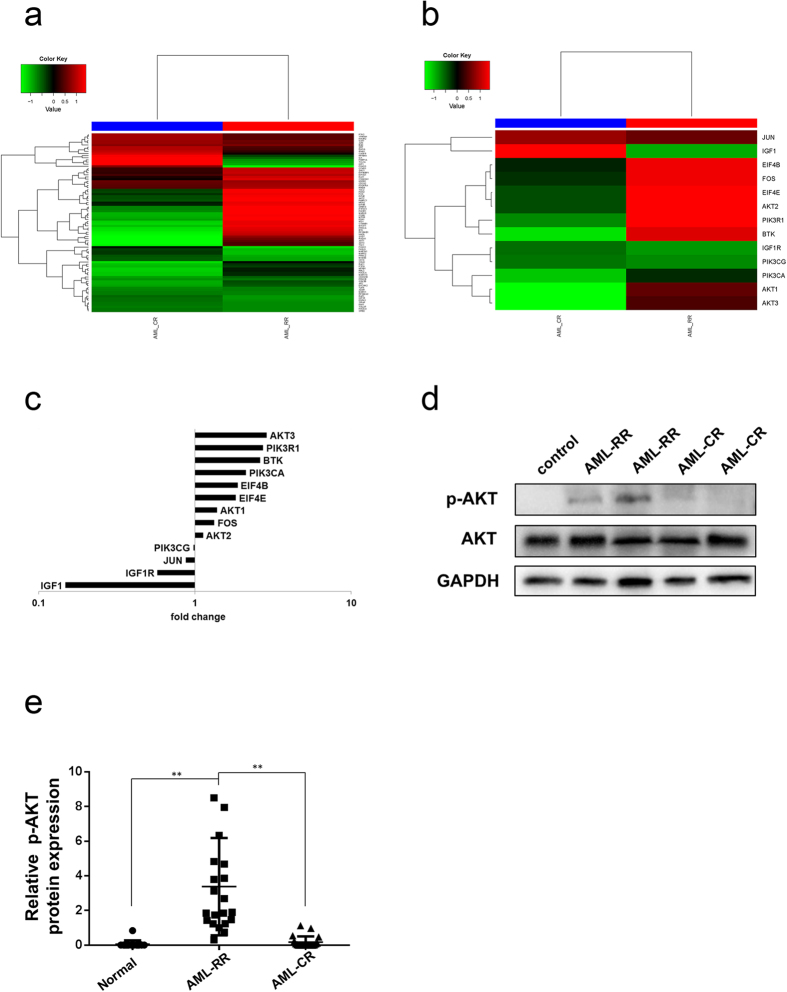
Gene expression detected by RNA-Seq and expression of p-AKT in AML patients. (**a**) Heatmap of PI3K/AKT-related genes. (**b**) Heatmap of selected PI3K/AKT-related genes. (**c**) Fold-change in the expression of selected PI3K/AKT-related genes in AML-CR and AML-RR patients. (**d**,**e**) Western blot analysis (**d**) and quantification of AKT and p-AKT protein expression (**e**) in AML-CR and AML-RR patients. *P < 0.05, **P < 0.01.

As previously noted, we also found that the majority of genes involved in the PI3K/AKT pathway, such as AKT1, AKT2, AKT3, and BTK (Fig. 2a–c, Table 3) were upregulated in the AML-RR group compared with the AML-CR group. Western blot validated the increased phosphorylation of AKT in the AML-RR group (Fig. 2d,e, P < 0.01). Together, these results suggested that the PI3K/AKT pathway is activated in the AML-RR group.

### Overexpression of GLI1 upregulated p-AKT and this effect is abolished by a GLI1 inhibitor in acute myeloid leukemia cell lines

**Figure 3:**
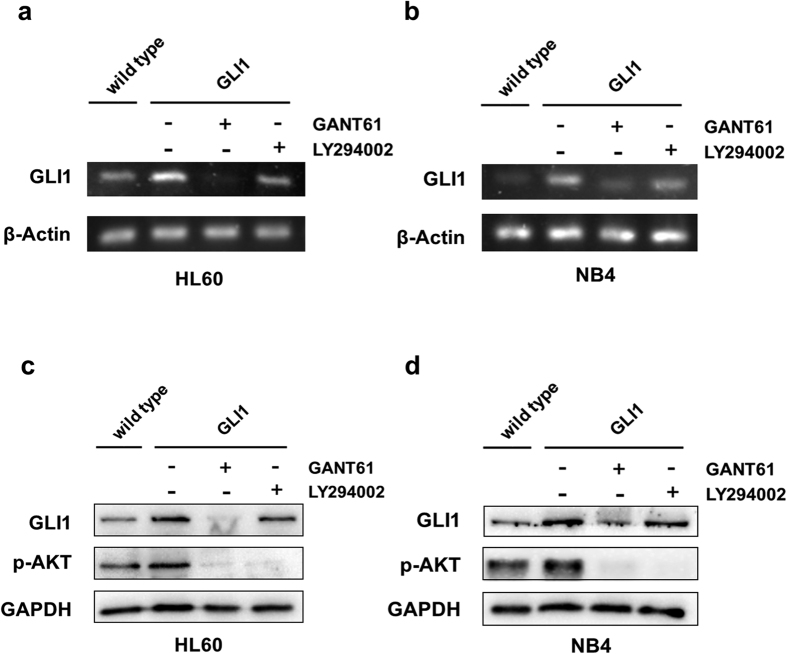
Expression of GLI1 and p-AKT in AML cell lines. (**a**,**b**) The transcript expression of GLI1 in both wild-type and GLI1 overexpressing HL60 cells (**a**) and NB4 cells (**b**) treated with 20 μM GANT61 or 20 μM LY294002. (**c**,**d**) The protein expression of GLI1 and p-AKT in both wild-type and GLI1 overexpressing HL60 cells (**c**) and NB4 cells (**d**) treated with 20 μM GANT61 or 20 μM LY294002. *P < 0.05, **P < 0.01.

To explore the role of GLI1 and the relationship of Hh and the PI3K/AKT pathway in AML relapse and drug resistance, GLI1 was overexpressed in both HL60 and NB4 cells by a lentiviral vector carrying EGFP for selection. Seventy-two hours after infection, cells were sorted by FACS to establish new cell lines, which were named HL60/GLI1 and NB4/GLI1. The upregulation of GLI1 in HL60/GLI1 and NB4/GLI1 was also confirmed by both RT-PCR (Fig. 3a,b) and western blot (Fig. 3c,d). Interestingly, overexpression of GLI1 increased the p-AKT level. GANT61, a GLI1 inhibitor, could reverse the increase in p-AKT by GLI1 overexpression, suggesting that GLI1 functions upstream of p-AKT. LY294002, a PI3K inhibitor, did not inhibit the expression of GLI1 (Fig. 3a–d). These results indicate that GLI1 could regulate the expression of p-AKT and had a unidirectional regulatory effect on the PI3K/AKT pathway.

### Inhibition of GLI1 could inhibit AML cell growth

**Figure 4:**
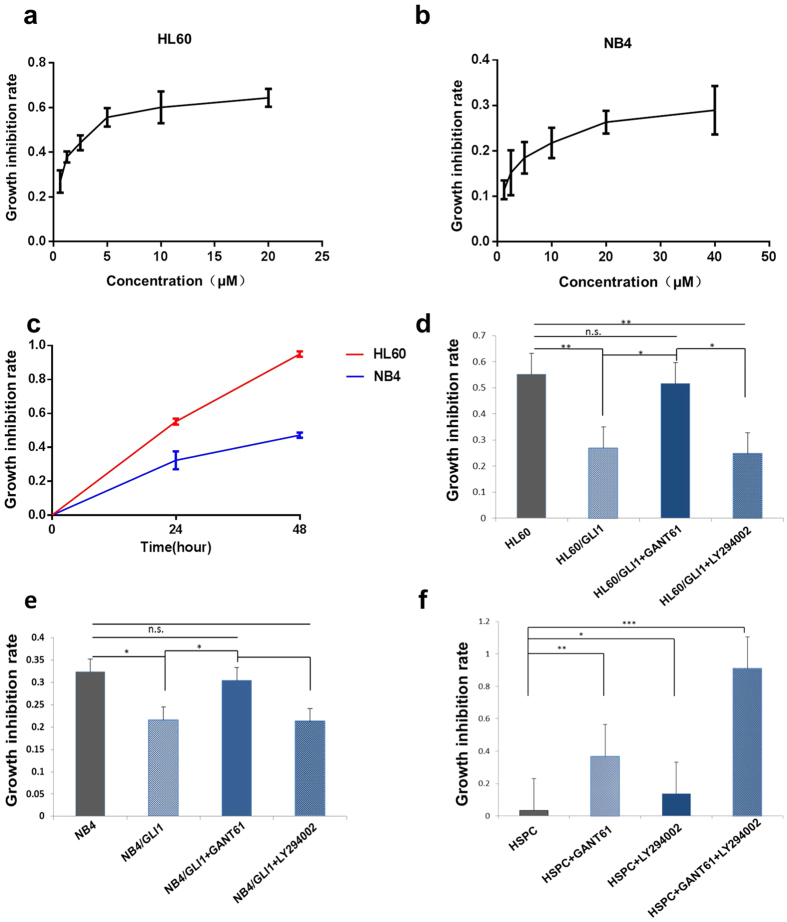
Cell proliferation of cell lines treated with Ara-C and GLI1 inhibitor or PI3K inhibitor. (**a**) Growth inhibition rate of HL60 cells with Ara-C, IC_50_ (half maximal inhibitory concentration) = 4 μM. (**b**) Growth inhibition rate of NB4 cells with Ara-C, IC_50_ = 25 μM. (**c**) Growth inhibitory curve of HL60 and NB4 cells in their IC_50_ values. (**d**) The growth inhibition rate in both wild-type and GLI1 overexpressing HL60 cells treated with Ara-C and GANT61/LY294002. (**e**) The growth inhibition rate in both wild-type and GLI1 overexpressing NB4 cells treated with Ara-C and GANT61/LY294002. (**f**) The growth inhibition rate in HSPCs treated with Ara-C (4 μM) and GANT61/LY294002. The results are shown as the mean of three independent experiments ± s.d. *P < 0.05.

Ara-C was assessed for its ability to inhibit AML cell growth and apoptosis. Ara-C inhibited the growth of HL60 and NB4 cells in a dose-dependent manner with an IC_50_ of 4 μM for the HL60 cells (Fig. 4a) and an IC_50_ of 25 μM for the NB4 cells (Fig. 4b). A time course experiment was used to optimize the timing and dose of Ara-C. HL60 and NB4 cells were treated with different concentration up to 48 h. The growth inhibition rate did not increase significantly when cells were treated with Ara-C at concentrations higher than 10 μM at both 24 h and 48 h (Fig. 4a–c). We found that the cell growth inhibition rates in both HL60/GLI1 and NB4/GLI1 cells were significantly lower than the wild-type cells. However, GANT61 treatment could compensate for this difference, suggesting that GLI1 is both sufficient and necessary to decrease drug sensitivity of Ara-C. Consistent with the previous results showing that LY294002 does not affect GLI1 expression, LY294002 inhibited neither HL60/GLI1 nor NB4/GLI1 cell growth (Fig. 4d,e). Besides, there was no difference in growth inhibition rate between the treatment of GANT61 or LY294002 in wild-type cells (Supplementary Fig. S4a). A major safety concern for AML is that the chemotherapy drugs also lead to severe bone marrow depression, which is related to the inhibition of proliferation in human HSPCs. Here, we tested the effect of GANT61 (20 μM), LY294002 (20 μM), and GANT61 + LY294002 (20 μM) on HSPCs. Surprisingly, the cell growth inhibition rate of the HSPCs in the GANT61 + LY294002 (20 μM) treatment group was higher than 90% (Fig. 4f), which raises the serious safety concern that the combination treatment may impair the human hematopoietic system.

### Inhibition of GLI1 could enhance acute myeloid leukemia cell drug sensitivity

**Figure 5:**
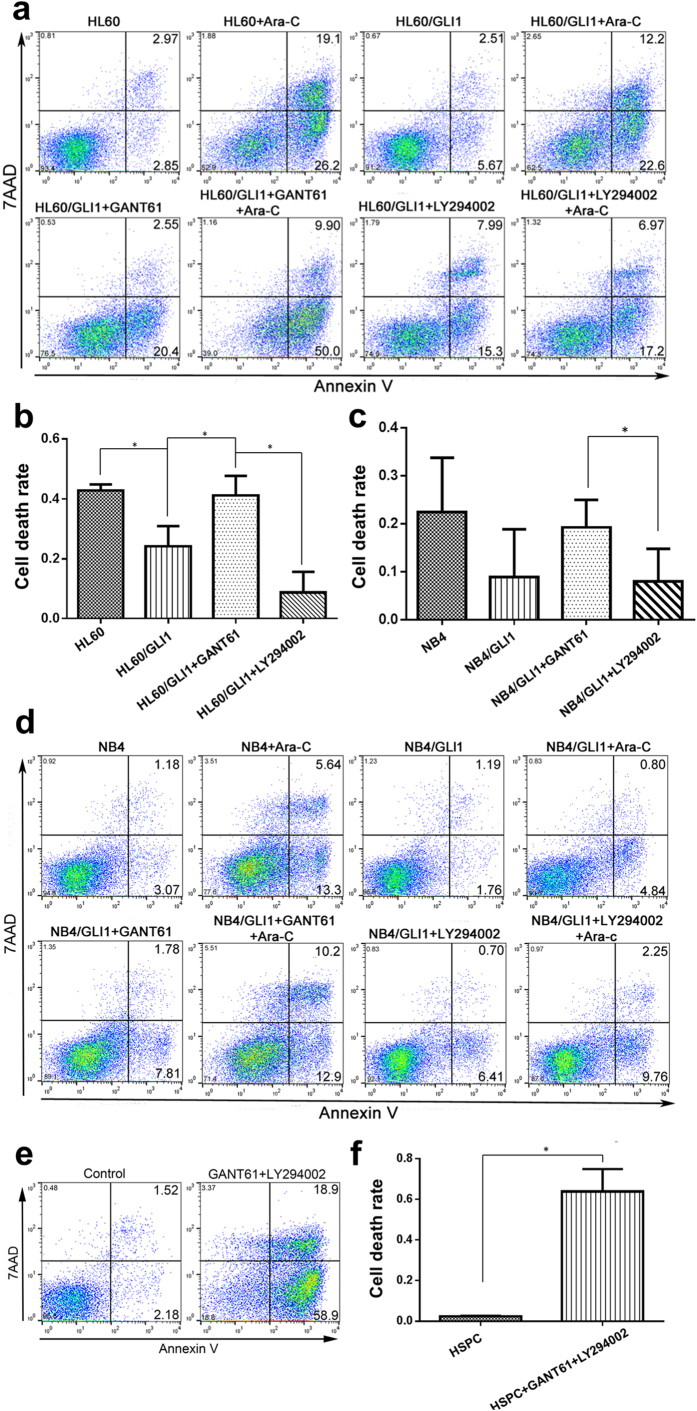
Cell apoptosis of HL60 and NB4 cells treated with Ara-C and GLI1 inhibitor or PI3K inhibitor. (**a**,**d**) Flow cytometry scatter plot of both wild-type and GLI1 overexpressing HL60 cells (**a**) or NB4 cells (**d**) treated with Ara-C and GLI1 inhibitor or the PI3K inhibitor. (**b**,**c**) Cell death rate in both wild-type and GLI1 overexpressing HL60 (**b**) or NB4 (**c**) cells treated with Ara-C and GLI1 inhibitor or PI3K inhibitor. (**e**,**f**) Flow cytometry scatter plot and cell death rate of HSPCs with both inhibitors of GLI1 and PI3K. *P < 0.05.

To test drug sensitivity, both wild-type and GLI1 overexpressing cells were treated with Ara-C only, Ara-C + GANT61 (20 μM), and Ara-C + LY294002 (20 μM) separately for 48 hours, and cell apoptosis was assessed using Annexin V assays (Fig. 5a,d). Consistent with the cell growth inhibitory results, GANT61 significantly enhanced the drug sensitivity of HL60 and NB4 cells, whereas LY294002 failed to do so (Fig. 5b,c). While, there was no difference in cell death rate between the treatment of GANT61 or LY294002 in wild-type cells (Supplementary Fig. S4b). Moreover, we found that the apoptosis rate of HSPCs with both inhibitors was significantly higher than that in the control group, which raises a serious concern about the safety of the combination treatment (Fig. 5e,f).

## Discussion

As the morbidity and mortality of leukemia are increasing every year, developing effective and safe treatment strategies is of great urgency. In recent years, targeted therapy, which is considered to be one of the most promising treatments for AML, has been extensively researched. However, only a few targeted drugs have been successful in clinical practice, such as all trans-retinoic acid and imatinib^24^,^25^,^26^. Therefore, new targets and associated drugs are necessary for future treatment of AML.

In this study, by comparing gene expression profiles of clinical bone marrow samples from AML-CR and AML-RR patients using RNA-Seq technology, we found that the Hh and PI3K/AKT pathways were upregulated in the AML-RR group. Consistent with the results of RNA-Seq, RT-PCR and western blot analyses also showed upregulated expression of GLI1 and p-AKT in the AML-RR patients. An *in vitro* study showed that inhibition of GLI1 depressed p-AKT activation and increased Ara-C sensitivity in leukemia cells, which is consistent with previous reports^12^,^15^,^27^. These results indicated that these pathways might play important roles in the progression and drug resistance of leukemia. Nevertheless, the expression of GLI1 in AML cells was not significantly affected by the PI3K inhibitor LY294002. Recently, several studies have reported that combined inhibition of the Hh and PI3K/AKT pathways can effectively inhibit the growth of cancer cells and has a synergistic anticancer effect on various tumours, such as rhabdomyosarcoma, pancreatic cancer, ovarian cancer and others^28^,^29^,^30^. These reports provided a basis for combination therapies targeting the Hh/GLI and PI3K pathways. However, the feasibility of clinical application has yet to be fully defined. In addition, our results showed that the both inhibitors had an irreversible and severe cytotoxicity to HSPCs, which raised the question of how to maximize the anti-leukemia effect of combined inhibition while minimizing the side effects to normal cells.

Other groups have shown that the AKT1 promoter possesses two GLI1 binding sites (BS1 and BS2) and proved the expression of AKT1 was regulated at the transcriptional level by GLI1^31^. Our results suggested that for AML patients with both GLI1 and AKT activation, the GLI1 inhibitor alone is sufficient, which will significantly decrease the unnecessary side effects of the combination therapy using Hh and PI3K inhibitors. The GLI1 inhibitor would provide a potential targeted therapy to AML cells, which have been reported in other hematonosis^32^. Additional studies are needed to identify the feasibility of clinical application by using GLI1 inhibitor in refractory/relapse population of AML patients.

Our study characterizes the Hh and PI3K/AKT interaction in AML drug sensitivity, which may provide a theoretical basis for further research of targeted therapy.

## Materials and Methods

### Cell culture and transfection

HL60 and NB4 cells were obtained from the Cell Resource Centre (Xiangya Medical College, Central South University, Hunan, China). The cell lines were maintained in RPMI-1640 medium (Corning Inc., Corning, NY, USA) containing 10% foetal bovine serum (Corning Inc.) and 1% antibiotic solution of penicillin/streptomycin (Sigma, MO, USA) in a 37 °C incubator with a humidified atmosphere of 5% CO_2_ (Supplementary Fig. S2). To overexpress GLI1, AML cells were infected with lentivirus containing the GLI1 open reading frame (MOI: 50–100). After 72 h of infection, HL60 and NB4 lines were allowed to recover for 24 h with fresh media and were referred to as HL60/GLI1 and NB4/GLI1 cells (Supplementary Fig. S3). To evaluate the response to drug treatment, 1 × 10^6^ HL60/GLI1 and NB4/GLI1 cells were seeded in 6-well culture plates and incubated with either 20 μM GANT61 (Adooq Bioscience, A13252) or 20 μM LY294002 (Adooq Bioscience, A10547) for 72 h prior to subsequent measurements.

### Patient characteristics

Liquid bone marrow samples from 42 patients between 18 and 64 years old, who were diagnosed with AML according to the 2008 WHO criteria and treated at Xiangya Hospital of Central South University, Hunan, China, were included in the AML group (Tables 1 and 2, Supplementary Fig. S1). For comparison, 15 healthy candidate donors were included as normal controls. The experimental protocols were approved by the ethical committee of Xiangya Hospital, Central South University. The informed consent was obtained from all the research subjects. All the bone marrow samples were collected into sterile tubes with anticoagulant (heparin sodium). Mononuclear cells (MNCs) were enriched by density centrifugation over Ficoll-Paque (Sigma, St. Louis, MO, USA) and stored at −80 °C.

### RNA-Seq

RNA sample quality was analysed, and the cDNA libraries were synthesized and sequenced using BGI technology. In brief, the quality of the RNA samples was assessed by an Agilent Bioanalyzer (Agilent). cDNA libraries were generated using TruSeq RNA Sample Preparation (Illumina). Each library was sequenced using single-reads on a HiSeq2000/1000 (Illumina). Gene expression levels were measured in RPKM using Cufflinks^33^,^34^.

### Quantitative RT-PCR and RT-PCR

RNA was isolated from 1 × 10^6^ cells using TRIzol reagent (TaKaRa, Japan, Cat#9109). Total RNA (500 ng) was reverse transcribed into first strand cDNA using a PrimeScript^TM^ RT Reagent Kit (TaKaRa, Japan, Cat#RR047A). cDNA was amplified in a total volume of 10 μl using SYBR qPCR Mix (Toy-obo, TOYOBO, Japan, Lot:266400), and the amplification was performed by ABI StepOnePlus (Applied Biosystems, Foster City, CA, USA) with specific primers (Table 4). The thermal cycler conditions were as follows: 95 °C for 2 min, followed by 40 cycles of 95 °C for 10 s, 58 °C for 20 s, and 68 °C for 50 s.

### Western blot analysis

Equal amounts of protein were solubilized in sample buffer and electrophoresed on denaturing SDS-polyacrylamide gels and then transferred to polyvinylidene fluoride (PVDF) membranes (Millipore, Billerica, MA, USA). The membranes were saturated in TBST containing 5% BSA (Bio Sharp Sigma A-4612) for an hour at room temperature and incubated with the primary antibodies overnight at 4 °C. After incubation with secondary antibody, the blots were then washed and detected with a ChemiDoc MP System (Bio-Rad Laboratories. Inc., Hercules, CA, USA). The antibodies were used as follows: mAb rabbit anti-GLI1 (C68H3) (Cell Signaling, 3538), mAb rabbit anti-phospho-AKT (Ser473) (Cell Signaling, 4060), mAb rabbit anti-AKT (Cell Signaling, 9272), anti-GAPDH (Santa Cruz, CA, USA), and horseradish peroxidase (HRP)-conjugated goat anti-rabbit IgG (Santa Cruz, CA, USA).

### CCK8 assay

The inhibition effect of cell proliferation was measured by a Cell Counting Kit-8 assay (7sea biotech, China) using different concentrations of Ara-C (Solarbio, Lot. No. 317B002) according to the protocol provided by the manufacturer. Then, 2 × 10^4^ cells were seeded in 96-well culture plates in 100 μL of medium per well and treated with Ara-C at IC_50_ (half maximal inhibitory concentration) and GANT61/LY294002 (20 μm) for 24 h. Finally, 10 μl CCK8 solution was added to each well, and the cells were incubated further for 3 h at 37 °C. Cell viability was evaluated based on the absorbance at 450 nm compared with 630 nm. The growth inhibition rate = (1 − OD value of treatment/OD value of control) × 100%.

### Annexin V APC assay

In each well, 1 × 10^6^ cells were seeded in 6-well culture plates and treated with Ara-C at its IC_50_ and GANT61/LY294002 (20 μm). After 24 h of culture, cells were analysed by flow cytometry with an Annexin V-APC kit (Beyotime Institute) to determine the cell death rate.

### Statistical analysis

All experiments were repeated independently at least three times, and the data were represented as the mean ± SD of the vehicle controls. All groups were compared using the Statistical Package for the Social Sciences (SPSS) version 20.0. The differences between the populations were assessed by analysis of covariance (ANCOVA). The diagrams were generated by GraphPad Prism 6 software. Significance was defined as *P < 0.05, **P < 0.01, ***P < 0.001, and ****P < 0.0001.

### Ethical approval

The experimental protocols and methods were performed in accordance with relevant guidelines and regulations. The informed consent was obtained from all the research subjects.

## Additional Information

**How to cite this article**: Liang, H. *et al*. Targeting the PI3K/AKT pathway via GLI1 inhibition enhanced the drug sensitivity of acute myeloid leukemia cells. *Sci. Rep.* **7**, 40361; doi: 10.1038/srep40361 (2017).

**Publisher's note:** Springer Nature remains neutral with regard to jurisdictional claims in published maps and institutional affiliations.

## Supplementary Material

Supplementary Information

## Figures and Tables

**Table 1:** Patients’ information.

	Patients,n	
Patient’s characteristics	AML-RR (n = 21)	AML-CR (n = 21)
Median age in years (range)	43.8 (22–59)	42.6 (18–64)
Sex (male/female)	13 (61.9%)/8 (38.1%)	10 (47.6%)/11 (52.4%)
Unfavorable fusion gene	9 (42.9%)	2 (9.5%)
Unfavorable karyotype	6 (28.6%)	2 (9.5%)

**Table 2:** Characteristics of the 42 patients.

Characteristic	Value
**Bone marrow blasts at diagnosis (%)**	59.94
AML FAB subtype (n,%)	
M1	1 (2.4%)
M2	18 (42.9%)
M3	2 (4.8%)
M4	11 (26.1%)
M5	6 (14.3%)
unidentified type	4 (9.5%)
Mutation — no./total no.(n,%)	
WT1	6 (14.3%)
FLT-ITD	2 (4.8%)
CEBPA	3 (7.1%)
NPM1	1 (2.4%)
EVI1	2 (4.8%)
JAK2	1 (2.4%)
DNMT3A	1 (2.4%)
MLL/AF9	1 (2.4%)
t (8;21)	2 (4.8%)

**Table 3:** Gene expression detected by RNA-Seq.

pathway	related genes
Hedgehog	DHH, BMP5, WNT9A, BMP2, BMP4, PTCHD1, FKBP8, WNT10A, WNT7A, WNT1, BMP8B, FGF9, WNT10B, PTCH2, IFT52, CSNK1E, WNT11, BTRC, WNT5A, RAB23, FGFR3, BOC, WNT8B, WNT5B, VEGFA, SMO, TP53, GAS1, HHIP, BCL2, GLI, ZIC2, ZIC1, HHAT, PRKACB, WNT6, SHH, WNT3, STK36, DISP2, WNT2, DISP1, PTCHD2, RUNX2, LRP2, BMP7, FBXW11, WNT4, SUFU, WNT2B, CSNK1A1, GSK3B, NPC1, KCTD11, WNT16, WNT3A, FOXE1, WNT8A, PTCH1, PRKACA, MTSS1, CDON, BMP6, MAPK1, WNT7B, NUMB, WNT9B, PTCHD3, OTX2, IHH, ERBB4
PI3K/AKT	PTK2, YWHAH, PIK3R2, GJA1, JUN, BAD, SRF, RHOA, IRAK1, CASP9, NFKBIA, ILK, FKBP1A, TCL1A, IGF1, FOXO3, CCND1, SHC1, EIF4EBP1, EIF4G1, ELK1, GRB2, CSNK2A1, HSPB1, CDC42, PDGFRA, RHEB, TSC2, PDK2, FOS, EIF4E, AKT2, PABPC1, HRAS, EIF4B, GRB10, PTPN11, PIK3R1, MAPK8, ITGB1, MTOR, PDK1, IRS1, CTNNB1, MTCP1, PRKCA, BTK, RPS6KB1, WASL, SOS1, RASA1, AKT1, AKT3, TSC1, FOXO1, FASLG, MAP2K1, MAPK3, PRKCZ, MYD88, CD14, CHUK, RAC1, PTEN, NFKB1, RBL2, PIK3CA, MAPK14, CDKN1B, PRKCB, GSK3B, APC, EIF2AK2, TLR4, ADAR, MAPK1, RPS6KA1, RAF1, IGF1R, PDPK1, TIRAP, PAK1, TOLLIP, PIK3CG,

**Table 4:** PCR primers and conditions.

Gene	Strand	Primer sequences	Annealing temperature
GLI1	Sense	5′-AACGCTATACAGATCCTAGCTCG-3′	58 °C
	Anti-sense	5′-GTGCCGTTTGGTCACATGG-3′	
β-Actin	Sense	5′-TGACGTGGACATCCGCAAAG-3′	58 °C
	Anti-sense	5′-CTGGAAGGTGGACAGCGAGG-3′	
