# Supplementary material for: Targeting the PI3K/AKT pathway via GLI1 inhibition enhanced the drug sensitivity of acute myeloid leukemia cells
Source: Sci Rep. 2017 Jan 18;7:40361. doi: 10.1038/srep40361 (PMC5241777; doi:10.1038/srep40361)
Supplement: Supplementary Information [file srep40361-s1.pdf]

Targeting the PI3K/AKT pathway via GLI1 inhibition enhanced the drug sensitivity of acute myeloid leukemia cells

Hui Liang[1], Qi-Li Zheng[1], Peng Fang[1], Jian Zhang[2], Tuo Zhang[7], Wei Liu[5], Min Guo[6], Christopher L. Robinson[4], Shui-bing Chen[4], Xiao-Ping Chen[3, 8*], Fang-Ping Chen[2*], and Hui Zeng[1*]

[1] Department of Hematology, Xiangya Hospital, Central South University, Changsha, Hunan, China; [2] Department of Hematology, Third Xiangya Hospital, Central South University, China; [3] Department of Clinical Pharmacology, Xiangya Hospital, Central South University, China; [4] Department of Surgery Genomic Core, Weill Cornell Medical College, NY, USA; [5] Department of Oncology, Xiangya Hospital, Central South University, China; [6] Department of Endocrinology, Xiangya Hospital, Central South University, China; [7] Genomic Core, Weill Cornell Medical College, NY, USA; [8] Institute of Clinical Pharmacology, Central South University, China.

Supplementary Figures with captions

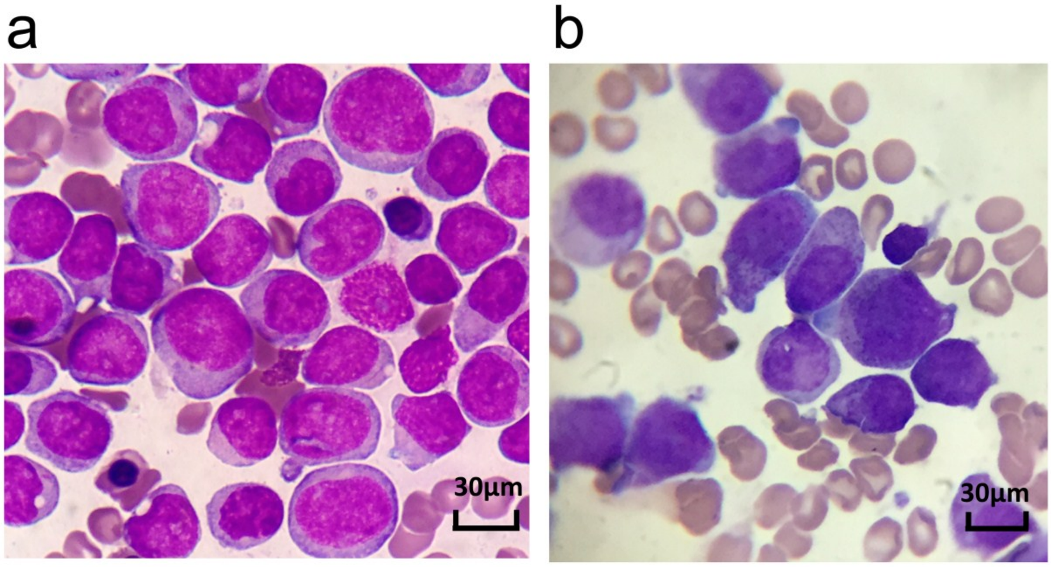

Figure S1. Bone marrow biopsies for AML patients (Wright–Giemsa staining).10×100 (a) Bone marrow biopsy for M2 AML patient. (b) Bone marrow biopsy for M3 AML patient.

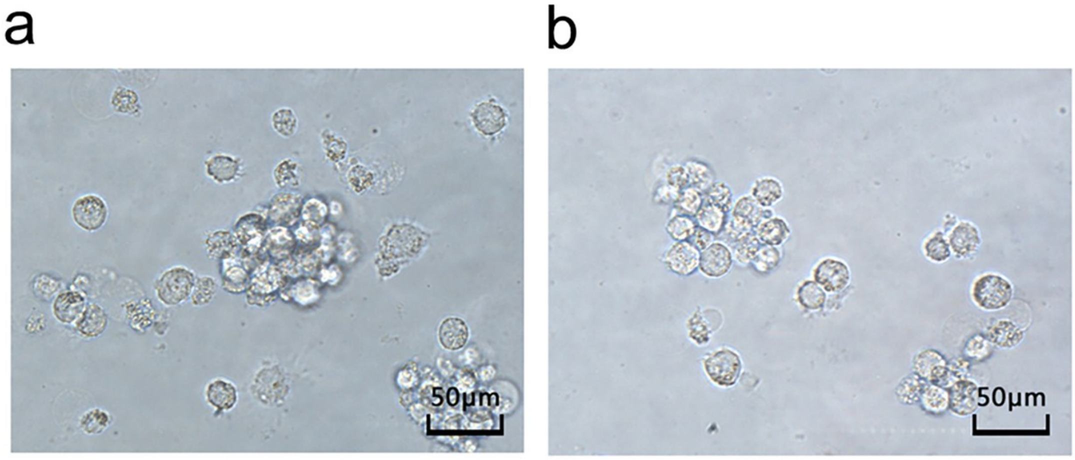

Figure S2. Growth of HL60 (a) and NB4 (b) cells.10 × 40

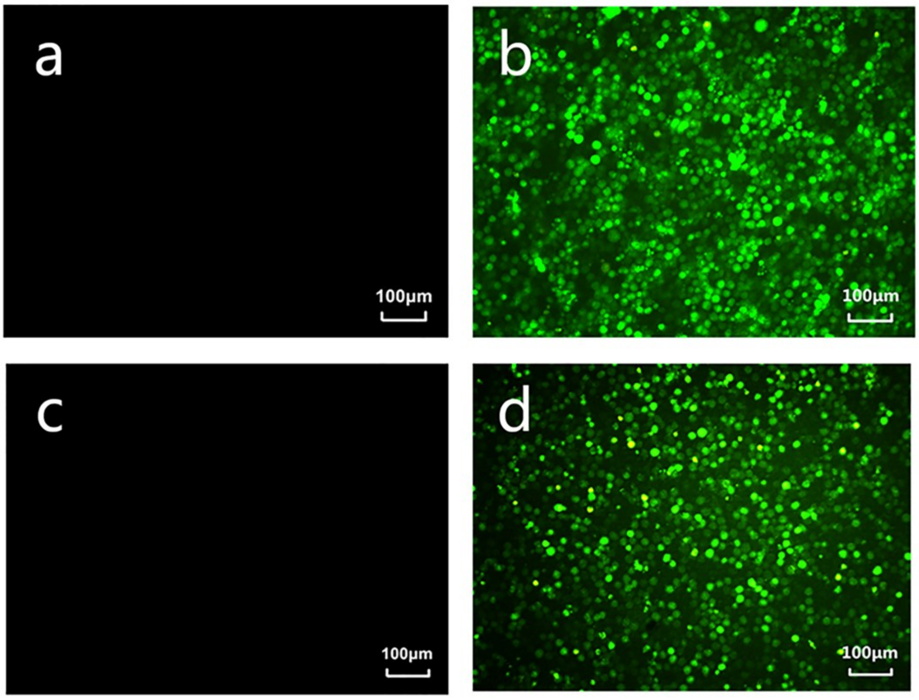

Figure S3. Growth of HL60 (ab) cells and NB4 (cd) under an inverted microscope by using fluorescence microscopy after transfection for 72h. 10 × 10

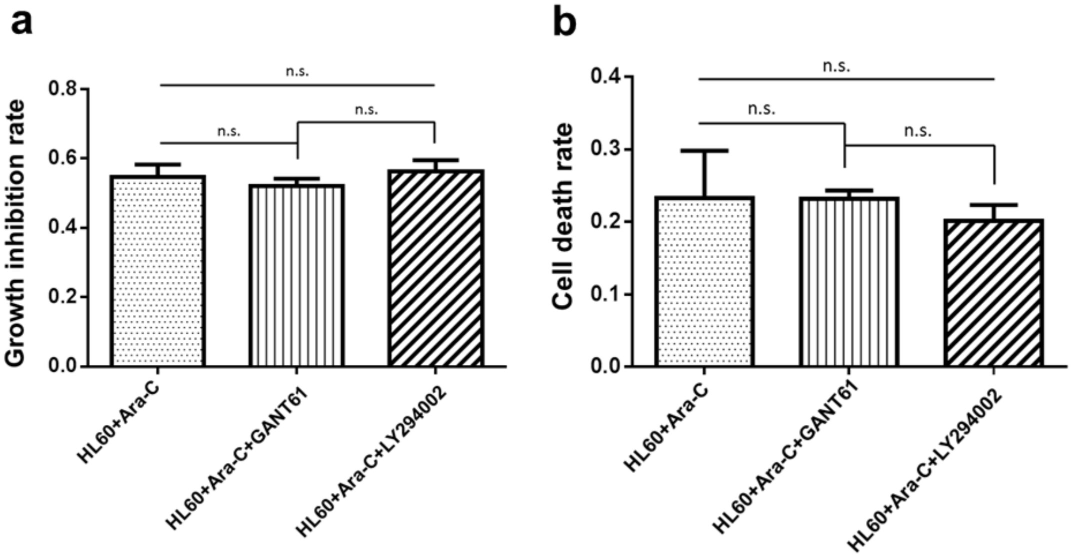

Figure S4. The growth inhibition rate (a) and cell death rate (b) in wild-type HL60 cells treated with Ara-C and combined with GANT61 or LY294002
